# Proportion of pregnant women screened for hypertensive disorders in pregnancy and its associated factors within antenatal clinics of Kinshasa, Democratic Republic of Congo

**DOI:** 10.1186/s12884-019-2435-z

**Published:** 2019-08-15

**Authors:** Dalau Mukadi Nkamba, John Ditekemena, Gilbert Wembodinga, Pierre Bernard, Antoinette Tshefu, Annie Robert

**Affiliations:** 1 Kinshasa School of Public Health, Faculty of Medicine, University of Kinshasa, Kinshasa, Democratic Republic of Congo; 2 Institut de Recherche Expérimentale et Clinique (IREC), Pôle d’Epidémiologie et Biostatistique (EPID), Université catholique de Louvain (UCLouvain), Brussels, Belgium; 3 Institut de Recherche Expérimentale et Clinique (IREC), Département d’obstétrique, Saint-Luc University Hospital, Université catholique de Louvain (UCLouvain), Brussels, Belgium

**Keywords:** Screening, Hypertensive disorders in pregnancy, Kinshasa

## Abstract

**Background:**

Screening for hypertensive disorders in pregnancy (HDP) is clinically important for identifying women at high risk, and planning early preventative interventions to improve pregnancy outcomes. Several studies in developing countries show that pregnant women are seldom screened for HDP. We conducted a study in Kinshasa, DR Congo, in order to assess the proportion of pregnant women screened for HDP, and to identify factors associated with the screening.

**Methods:**

We conducted a facility-based cross-sectional study in a random sample of 580 pregnant women attending the first antenatal visit. Data collection consisted of a review of antenatal records, observations at the antenatal care services, and interviews. A pregnant woman was considered as screened for HDP if she had received the tree following services: blood pressure measurement, urine testing for proteinuria, and HDP risk assessment. Multivariable logistic regression, with generalized estimating equations, was used to identify factors associated with the screening for HDP.

**Results:**

Of the 580 pregnant women, 155 (26.7%) were screened for HDP, 555 (95.7%) had their blood pressure checked, 347(59.8%) were assessed for risk factors of HDP, and 156 (26.9%) were tested for proteinuria. After multivariable analysis, screening for HDP was significantly higher in parous women (AOR = 2.09; 95% CI, 1.11–3.99; *P* = 0.023), in women with a gestational age of at least 20 weeks (AOR = 5.50; 95% CI, 2.86–10.89; *P* = 0.002), in women attending in a private clinic (AOR = 3.49; 95% CI, 1.07–11.34; *P* = 0.038), or in a hospital (AOR = 3.24; 95% CI, 1.24–8.47; *P* = 0.017), and when no additional payment was required for proteinuria testing at the clinic (AOR = 2.39; 95% CI, 1.14–5.02; *P* = 0.021).

**Conclusion:**

Our results show that screening for HDP during the first antenatal visit in Kinshasa is not universal. The factors associated with screening included maternal as well as clinics’ characteristics. More effort should be made both at maternal and clinic levels to improve the screening for HDP in Kinshasa.

## Background

Hypertensive disorders in pregnancy (HDP) are the second most common cause of maternal mortality worldwide [1]. Most deaths are avoidable and occur in low- and middle-income countries (LMIC), due to a lack or a delay in identifying women at high-risk, and in their treatment [2–5]. HDP screening is an essential element of antenatal care (ANC) that allows the identification of women at high risk of developing HDP or HDP-related complications, and the implementation of preventive interventions for improving perinatal outcomes [6–12]. Screening for HDP at the first antenatal visit includes blood pressure measurement, urine testing for proteinuria, and identification of maternal risk factors [13–17]. The National Institute for Health and Clinical Excellence (NICE) and the American College of Obstetricians and Gynecologists (ACOG) have provided a list of maternal risk factors to be assessed at the first antenatal visit [13, 18]. To prevent HDP in LMIC, where biomarkers are not available to identify women at high risk, the International Society for the Study of Hypertension in Pregnancy (ISSHP) recommends the use of low dose aspirin started before 16 weeks of pregnancy in women with any of the following risk factors: previous preeclampsia, chronic hypertension, underlying renal disease, diabetes mellitus, obesity, and antiphospholipid antibody syndrome [17].

In sub-Saharan Africa, while blood pressure is measured in more than half of antenatal attendees, proteinuria testing is performed less frequently [19–23]. Studies report 10% of women tested for proteinuria in Mozambique [21], 23% in Zambia 23% [19], and 32% in Ethiopia [24]. In a study in six sub-Saharan Africa countries including Tanzania and Rwanda, only 46% of women had a urine test for proteinuria [25]. According to the Demographic and Health Survey (DHS) conducted in 2014 in Democratic Republic of Congo (DRC), urine samples were collected during antenatal visits in 94.1 and 53.1% of women who delivered in the five years preceding the survey in Kinshasa and DRC, respectively [26]. However, laboratory tests performed on these urine samples were not specified. According to the World Health Organization (WHO), the maternal mortality is estimated at 693 deaths per 100,000 live births in the DRC [27]. This is higher than one might expect based on a high rate of antenatal care attendance (89%) and a high rate of skilled attendance at delivery(80%) [26]. According to the DRC’s National Health Information System (NHIS), 561 (52%) of the 1088 cases of preeclampsia reported in Kinshasa in 2017 developed eclampsia [28], making HDP the major cause of adverse pregnancy outcomes. During the first six months of 2018, HDP accounted for 841 (23%) of the 3656 maternal deaths that were reported to the DRC’s maternal death surveillance system [29]. Given that HDP are the second most common cause of maternal mortality in DRC [29], preventing HDP and HDP-related adverse pregnancy outcomes can contribute to the attainment of Sustainable Development Goal 3, which aims to reduce the maternal and neonatal mortality [30]. To our knowledge, no study to date has evaluated the screening for HDP during antenatal care in Kinshasa. We conducted the current study to determine the proportion of pregnant women screened for HDP during their first ANC visit in Kinshasa, and to identify factors associated with HDP screening.

## Methods

### Study setting

The study was carried out in 58 clinics that participated in the HDP Service Availability and Readiness Assessment (SARA) study that was previously conducted in Kinshasa. These 58 clinics (30 primary, 26 secondary and 2 tertiary) were selected using the stratified random procedure from a sampling frame of 837 primary, 138 secondary and 2 tertiary facilities that provide emergency obstetric and neonatal care (EmONC). In the DRC’s tierced health system, primary health centres (PHCs) provide basic curative and preventive services. The district hospitals (or Referral Health Centres where no district hospitals exist) are secondary level facilities, providing comprehensive emergency obstetric and neonatal care, and represent referral facilities for PHCs. Tertiary facilities include provincial reference hospitals and teaching hospitals. However, some PHCs refer directly to tertiary-level facilities [31].

### Study design, population, and sample size

We conducted a facility-based cross-sectional study among pregnant women attending the first ANC visit in the 58 above mentioned clinics. The sample size was obtained by using the formula for a single population proportion. Using a non-informative prior of 50% for the proportion of women screened for HDP, a margin error of 5%, a non-response rate of 10%, and a design effect of 1.3 based on our pilot study, a sample of 555 pregnant women was required. This sample size was divided by 58 (number of clinics) to get a minimum number needed per clinic, i.e. 555/58 = 9.6, which was rounded up to 10, leading to a final sample size of 580 women.

### Sampling procedure

For each clinic, we estimated the average number of first ANC visits per month (Yi; i stands for clinic number) from the most recent quarterly report of the clinic. This average number of first ANC visits (Yi) was considered as a sampling frame. We calculated Xi, the sampling step, by dividing Yi by 10, because 10 was the targeted sample size for each clinic. A number between 1 and Xi, called Wi hereafter, was then chosen at random with a uniform generator. In each clinic, all women presenting for a first ANC visit were consecutively numbered. The pregnant woman presenting with the number Wi in the sequential list of consultations was kept as the first one in our sample. Then, the 9 remaining pregnant women were systematically chosen at each Xi step in the list of consultations. In other words, all women numbered Wi + k*Xi (for k = 0 to 9) were sampled among the consecutive list of those who presented at the ANC service during the one-month period of data collection.

### Operational definitions

We used the following definitions in assessing the screening for HDP, and analyzing results:

#### HDP screening package

The entire HDP screening package consists of 1) blood pressure measurement, 2) urine test for proteinuria, and 3) HDP risk assessment.

#### Risk of HDP

Known risk factors for HDP including a history of diabetes, HDP in a previous pregnancy (if parous), a history of chronic hypertension, a history of renal disease.

#### HDP risk assessment

Enquiring about at least one of the following risk factors: HDP in a previous pregnancy (if parous), a history of chronic hypertension, a history of diabetes, a history of renal disease.

#### Woman screened for HDP

A pregnant woman who had received the entire HDP screening package during the ANC visit.

#### Late ANC booking

First ANC visit beyond 16 weeks of pregnancy.

### Data collection

Twelve physicians were recruited as surveyors, based on their previous experience in data collection, and two health officers as supervisors. Surveyors and supervisors were trained during five days before data collection. The training addressed the aim of the study, all procedures, and data collection techniques. We used a study questionnaire on maternal socio-demographic and obstetrical characteristics, and on clinics’ and ANC providers’ characteristics. A checklist for direct observation of an antenatal consultation was also used. This checklist focused on the screening for HDP. For each selected pregnant woman, a surveyor observed ANC provider conducting ANC consultation. He checked whether the ANC provider enquired about risk factors for HDP, whether he measured blood pressure, and whether he performed urine test for proteinuria. When the ANC consultation was completed, surveyors directly interviewed the woman about her socio-demographic and obstetrical data. Prior to the data collection, the questionnaire was pilot-tested in 10 clinics not included in this study. Data were collected between October 2017 and January 2018.

### Data analysis

Data were entered into EpiData software version 3.1 database and subsequently exported in Stata 14 for statistical analyses. We computed normalized weights to account for the unequal inclusion probability of pregnant women. The weights were obtained by inverting the inclusion probability of pregnant women. The inclusion probability of a pregnant woman was obtained by dividing 10 by the average number of pregnant women per month in the clinic. Weights were then normalized to set the weighted sample size to 580. To obtain normalized weights, we multiplied the weights by the unweighted sample size (*n* = 580) and divided by the sum of weights. All analyses were weighted using normalized weights. There was no missing data. During descriptive analysis, categorical variables were summarized using weighted proportions. Continuous variables were summarized using weighted mean and standard deviation (SD) if normal distributed, or median and interquartile range (IQR) otherwise. Secondary and tertiary health facilities were grouped into one category named “hospitals”, as they all represent referral units for PHCs.

We computed weighted proportions of pregnant women who received an antenatal service by type of facilities (PHCs or hospitals; or private and public), and by gestational age (less than 16 weeks, and 16 weeks and above), and compared them using a weighted chi square test.

Our dependent variable was binary (screened for HDP, yes or no). We used logistic regression analysis, with generalized estimating equations (GEE), to control for correlation among pregnant women at the same clinic. All variables with a *P*-value less than 0.25 in simple regression were candidates for multivariable analysis. If a strong correlation was noticed between two explanatory variables, one of the two was eliminated to avoid multicollinearity. Multicollinearity among independent variables was checked using the variance inflation factor (VIF). A VIF larger than 10 was indicative of the multicollinearity [32]. Study results are presented as odds ratios (OR) or adjusted odds ratios (AOR) with 95% confidence intervals (95%CI). The statistical significance level was set to 0.05.

## Results

### Characteristics

Of the 58 clinics, 41 (70.7%) were private and 17 (29.3%) were public. The median number of pregnant women attended per month per clinic was 50 (IQR: 37 to 63) (Table 1). The median age of the 580 pregnant women was 28 (IQR: 23 to 33) years (range, 15 to 44 years). The majority of women (60%) reached at least a secondary level of schooling. Roughly 83% (95% IC, 78.9–86.9%) of women booked ANC beyond 16 weeks of pregnancy. In both PHCs and hospitals, women’s socio-demographic characteristics were comparable (Table 2).

**Table 1:** Characteristics of the 58 participating antenatal clinics in Kinshasa

Characteristics	
Number of pregnant women attended^a^—no./mth	
Median (IQR)	50(37–63)
Type—no. (%)	
Primary	30 (51.7)
Secondary	26 (44.8)
Tertiary	2 (3.5)
Ownership—no. (%)	
Private	41 (70.7)
Public	17 (29.3)
Funded—no. (%)	
Yes	35 (60.3)
No	23 (39.7)
Location—no. (%)	
Rural area	7 (12.1)
Urban area	51 (87.9)
Proteinuria test available^b^—no. (%)	
Yes	39 (67.2)
No	19 (32.8)
Additional payment required for proteinuria testing^c^—no. (%)	
Yes	50 (86.2)
No	8 (13.8)

Abbreviations: *IQR* Interquartile range, *mth* months; *no* number

^a^The mean number of pregnant women attended monthly per clinic

^b^Dipsticks or acetic acid available during the study period

^c^Whether women have to pay extra money to be tested

**Table 2:** Characteristics of interviewed pregnant women attending antenatal care in 30 Primary Health Centres, and 28 hospitals sampled in Kinshasa

Characteristics	Primary health centres (*n*^a^ = 260)	Hospitals (*n*^a^ = 320)	All (*n*^a^ = 580)
Maternal age—yrs			
Median (IQR)	27(23–33)	29(23–34)	28(23–33)
Age group—no. (%)			
15–19	32(12.4)	50(15.6)	82(14.1)
20–34	195(74.8)	197(61.6)	392(67.6)
35–44	33(12.8)	73(22.8)	106(18.3)
Marital status—no. (%)			
Married/in union	232(89.2)	271(84.7)	503(86.7)
Single/Separated	28(10.8)	49(15.3)	77(13.3)
Scholarship—no. (%)			
Illiterate	14(5.4)	2(0.6)	16(2.8)
Primary	106(40.8)	109(34.1)	215(37.1)
Secondary or technical	105(40.4)	139(43.4)	244(42.0)
University or high school	35(13.4)	70(21.9)	105(18.1)
Occupation—no. (%)			
Housewife	155(59.6)	176(55.0)	331(57.1)
With a salary	105(40.4)	144(45.0)	249(42.9)
Gestational age–weeks			
Median (IQR)	22(18–25)	23(18–27)	22(18–25)
Group—no. (%)			
< 16 weeks	43(16.5)	54(16.7)	97(16.9)
≥ 16 weeks	217(83.5)	266(83.3)	483(83.1)
Gravidity—no. (%)			
1	47(18.1)	80(25.0)	127(21.9)
2 to 4	175(67.3)	186(58.1)	361(62.2)
≥ 5	38(14.6)	54(16.9)	92(15.9)
Parity—no. (%)			
0	66(25.4)	89(27.8)	155(26.7)
1 to 3	168(64.6)	193(60.3)	361(62.2)
≥ 4	26(10.0)	38(11.9)	64(11.1)

Abbreviations: *yrs*. years; *IQR* Interquartile range

^a^: weighted number of pregnant women

### Screening status for HDP

Out of the 580 pregnant women, 155 (26.7%; 95% IC, 18.4–37.1%) were screened for HDP, 555 (95.7%; 95% IC, 90.1–98.2%) had their blood pressure checked, 347(59.8%; 95% IC, 49.6–69.2%) were assessed for risk factors of HDP, and 156 (26.9%; 95% IC, 18.6–37.4%) were tested for proteinuria (Table 3). The proportion of pregnant women whose blood pressure was measured was significantly higher in hospitals than in PHCs (99.4% vs 91.1%; *P* = 0.009). There were no significant differences between PHCs and hospitals in the proportion of women tested for proteinuria and those assessed for risk factors (Table 3).

**Table 3:** Weighted proportion of pregnant women who received antenatal service at the time of the survey, according to types of clinics^c^

Service received	All (*n* = 580)	Primary Health Centres (*n* = 260)	Hospitals (*n* = 320)	Weighted X^2^ *P*-value
Checking history of renal disease	7 (1.2)	3 (1.2)	4 (1.3)	0.91
Checking history of HDP in previous pregnancies^a^	103 (22.7)	50 (23.5)	53 (22.1)	0.89
Urine test for proteinuria	156 (26.9)	55 (21.2)	101 (31.6)	0.23
Checking history of diabetes	196 (33.8)	80 (30.8)	116 (36.3)	0.61
Checking history of hypertension	198 (34.1)	83 (31.9)	115 (35.9)	0.63
Assessing body mass index	248 (42.8)	89 (34.2)	159 (49.7)	0.12
Checking at least one risk factor^b^	347(59.8)	138 (53.1)	209 (65.3)	0.21
Blood pressure measurement	555(95.7)	237 (91.2)	318 (99.4)	0.009

Abbreviations: *HDP* Hypertensive disorders in pregnancy

^a^Only in 453 women with previous pregnancies: 213 in Primary Health Centres, 240 in hospitals;

^b^Checking at least one of the following risk factors: HDP in previous pregnancies (if parous), history of chronic hypertension, history of diabetes, and history of renal disease

^c^Data were weighted according to normalized weights

**Fig. 1:**
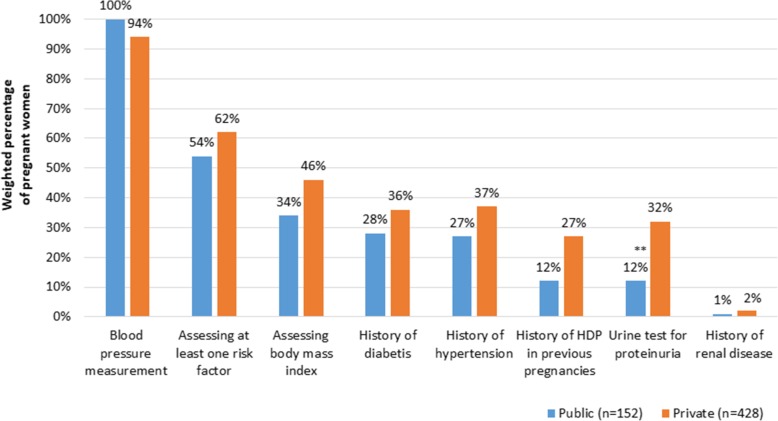
Weighted percentage of pregnant women who received a screening service at the time of the survey, in public as compared with private clinics. Weighted chi square test: **P < 0.01

In hospitals, blood pressure measurement was significantly less prevalent among women attending before 16 weeks of pregnancy, as compared to those attending at 16 weeks and above (*P* = 0.042). Both in PHCs and in hospitals, urine test for proteinuria was significantly less performed in women attending before 16 weeks, than those at at least 16 weeks (Table 4). There were significantly more women tested for proteinuria in private than in public clinics (32% vs 12%; *P* < 0.01). There was no significant difference in risk assessment and blood pressure measurement between public and private clinics (Fig. 1).

**Table 4:** Weighted proportion of pregnant women who received antenatal service at the time of the survey, in Primary Health Centres and in hospitals, according to the gestational age^b^

Service received	Primary health centers			Hospitals		
	Gestational age in weeks					
	< 16 weeks (*n* = 43)	≥16 weeks (*n* = 217)	Weighted X^2^ *P*-value	< 16 weeks (*n* = 53)	≥16 weeks (*n* = 267)	Weighted X^2^ *P*-value
Checking history of renal disease	2.2	0.9	0.001	1.4	1.2	0.89
Urine test for proteinuria	6.8	24.2	0.02	10.7	35.7	0.02
Checking history of HDP in previous pregnancies	15.7	24.8	0.102	8.9	24.7	0.033
Assessing body mass index	17.2	37.6	0.034	30.2	53.6	0.013
Checking history of hypertension	18.9	34.4	0.11	45.7	34.1	0.19
Checking history of diabetes	21.9	32.8	0.16	31.5	37.1	0.39
Checking at least one risk factor^a^	39.6	55.6	0.13	58.7	66.8	0.43
Blood pressure measurement	91.1	91.1	0.95	96.6	100.0	0.042

Abbreviations: *HDP* Hypertensive disorders in pregnancy, *X*^*2*^: Chi square

^a^Checking at least one of the following risk factors: HDP in previous pregnancies (if parous), history of chronic hypertension, history of diabetes, and history of renal disease

^b^Data were weighted according to normalized weights

**Fig. 2:**
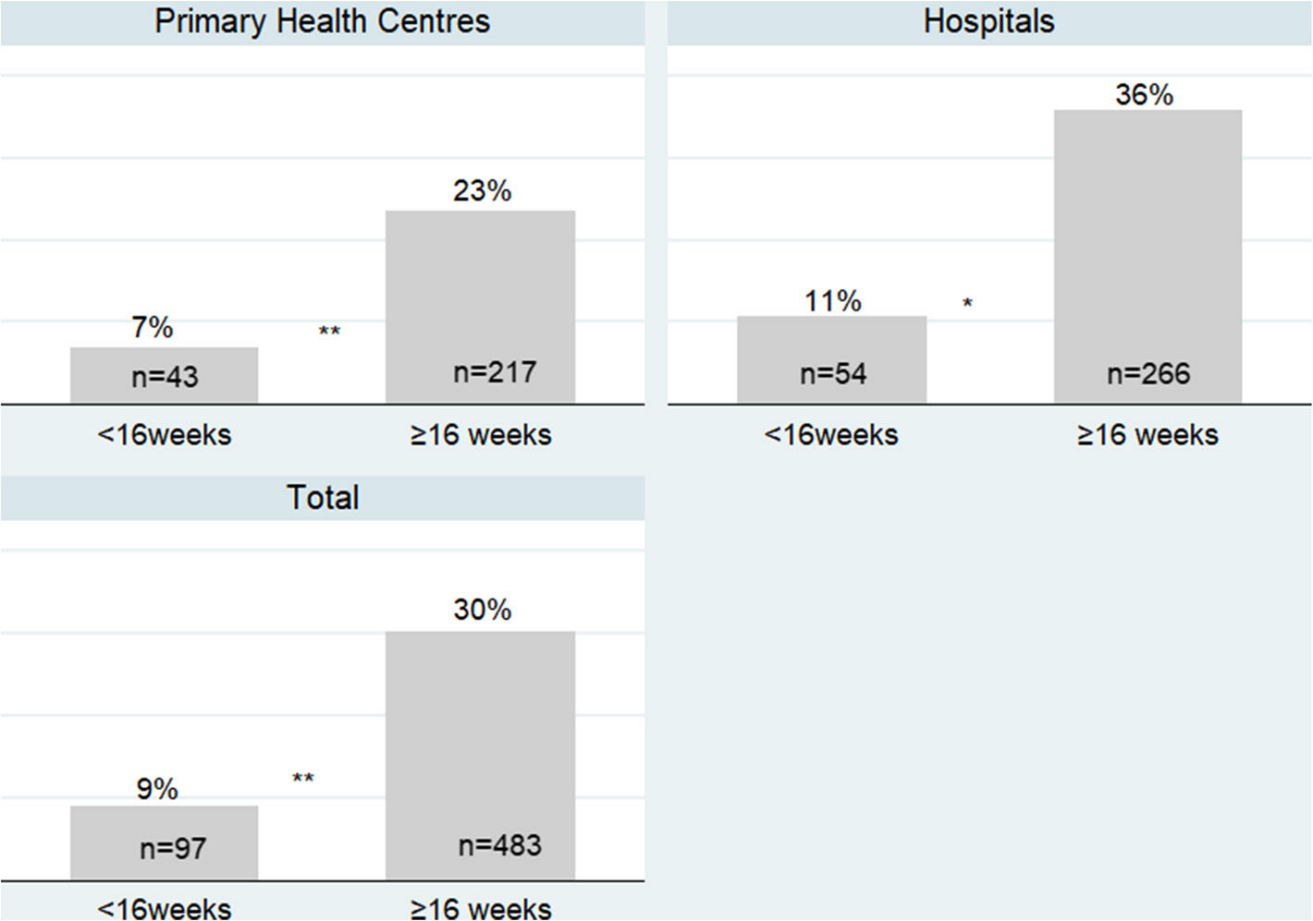
Weighted percentage of pregnant women screened for hypertensive disorders in pregnancy at the time of the survey, according to gestational age in weeks and types of clinics^¤.^ Weighted chi square test: **p < 0.01 *p < 0.05. ^¤^A pregnant woman was considered as screened for HDP if she received the following services: blood pressure measurement, urine test for proteinuria, and HDP risk assessment

Screening for HDP was significantly lower in women attending before 16 weeks, than in those attending at at least 16 weeks, both in PHCs and hospitals (Fig. 2).

### Factors associated with screening for HDPS

After multivariable analysis, screening for HDP was significantly higher in parous women (AOR = 2.09; 95% CI, 1.11–3.99; *P* = 0.023), in women with a gestational age of at least 20 weeks (AOR = 5.50; 95% CI, 2.86–10.89; *P* = 0.002), in women attending in a private clinic (AOR = 3.49; 95% CI, 1.07–11.34; *P* = 0.038), or in a hospital (AOR = 3.24; 95% CI, 1.24–8.47; *P* = 0.017), and when no additional payment was required for proteinuria testing at the clinic (AOR = 2.39; 95% CI, 1.14–5.02; *P* = 0.021) (Table 5).

**Table 5:** Factors associated with the screening for hypertensive disorders in pregnancy in 580 pregnant women attending antenatal care in 58 clinics of Kinshasa, using weighted GEE logistic regression model

Factors	no./N (%)	Crude OR (95% IC)	*P*-Value	Adjusted OR (95% IC)	*P*-Value
Parity			0.02		0.023
0	24/155 (15.5)	1		1	
≥ 1	131/426 (30.8)	1.84(1.11–3.04)		2.09(1.11–3.99)	
Maternal age—yr			0.036		–
15–19	11/82 (13.4)	1			
≥ 20	144/498 (28.9)	2.01(1.04–3.84)			
Marital status			0.35		–
Maried/in union	135/503 (26.8)	1.44(0.67–3.08)			
Single/separated	20/77 (25.9)	1			
Gestational age—wks			0.006		0.002
< 20	11/189 (5.8)	1		1	
≥ 20	144/391 (36.8)	4.24(2.39–7.50)		5.50(2.86–10.89)	
Schooling			0.11		0.55
Primary or less	41/231 (17.7)	1		1	
Secondary or more	114/349 (32.7)	1.66(0.90–3.06)		1.92(0.98–3.74)	
Occupation			0.93		–
Housewife	86/331 (25.9)	1			
Employed	69/249 (27.7)	1.02(0.73–1.42)			
Clinic ownership			0.002		0.038
Private	136/428(31.8)	3.31(1.38–7.87)		3.49(1.07–11.34)	
Public	19/152 (12.5)	1		1	
Type of the clinic			0.19		0.017
PHCs	54/260 (20.8)	1		1	
Hospitals	101/320 (31.6.)	1.76(0.75–4.13)		3.24(1.24–8.47)	
Funded clinic			0.93		–
Yes	99/373 (26.5)	1			
No	56/207 (27.1)	1.04(0.41–2.63)			
Women attended by health provider trained in HDP management			0.79		–
Yes	40/150 (26.7)	1			
No	115/430 (26.8)	1.01(0.49–2.53)			
Location			0.93		–
Rural area	10/44 (22.7)	1			
Urban area	145/536 (27.1)	1.16(0.39–3.8)			
Additional payment required for proteinuria testing			0.001		0.021
No	46/91 (50.5)	3.45 (1.64–7.22)		2.39 (1.14–5.02)	
Yes	109/489 (22.3)	1		1	

Abbreviations: *GEE* Generalized estimating equations, *OR* Odds ratio, *HDP* Hypertensive disorders in pregnancy; *yrs*. years, *wks* weeks, no number of women screened; N total number of women

## Discussion

Our study showed a low level of provision of screening for HDP in Kinshasa, with only 26.7% of women fully screened. Nearly all women had their blood pressure checked. The failure of complete HDP screening was a lack of ascertaining risk factors in 40%, and a lack of urine testing for proteinuria in 73%. The latter made the overall gap in the quality of screening so high. Screening for HDP was significantly higher in parous women, in women with a gestational age of at least 20 weeks, in women attending in a private clinic or in a hospital, and when no additional payment was required for proteinuria testing at the clinic.

The prevalence of blood pressure measurement in our study is consistent with those reported in Kenya (96%) and Nigeria (92%); but higher than the range of 45 to 89% reported in other developing countries such as Mozambique and Ethiopia [25, 33–35]. Our findings regarding proteinuria testing are similar to those reported in Rwanda (31%) and Madagascar (29%), but lower than those reported in Tanzania (40%), in Kenya (59%) and in Ethiopia (66%) [25]. Despite the availability of urine tests in two-thirds of health facilities in Kinshasa, we found a low provision of urine testing for proteinuria. This suggests that other factors were not captured by our study, including poor knowledge among ANC providers or a lack of consistent national guidelines for the screening for HDP [25, 36].

Risk factors of HDP can be ascertained by a simple anamnesis of a woman during her ANC consultation [17]. In our study, only 59.8% of pregnant women were assessed for risk factors of HDP. To assess risk factors, providers should be aware of them. In studies from Bangladesh and Nigeria, only 2 and 15% of ANC providers were aware of risk factors for HDP, respectively [37, 38]. The failure to assess women for risk factors of HDP in Kinshasa may be a consequence of providers’ knowledge gaps, but the point needs to be investigated.

The low provision of screening for HDP in Kinshasa (26.7%) may also be due to a lack of consistent national guidelines regarding the prevention of HDP, which can subsequently induce knowledge gaps among health providers [39]. This low provision of screening for HDP raises the issue of the quality of antenatal care services, and implies a missed opportunity to prevent HDP which account for up to 23% of maternal deaths in DRC [29].

Screening for HDP is critically important in PHCs, even if they are generally less equipped, because it allows timely referral of the woman to a higher level facility with more appropriate surveillance tools [2, 17, 40, 41]. Our findings indicate that women having ANC visits in PHCs were less likely to be screened than those in hospitals. This finding is consistent with other studies reporting also a low provision of maternal health services in PHCs compared to hospitals [42]. More efforts should be devoted to promoting systematic screening for HDP in PHCs as they represent the first contact health facility in Kinshasa.

Studies have shown the benefit of aspirin in the prevention of HDP in high-risk women when it is started before 16 weeks of pregnancy, or definitely no later than 20 weeks [6, 17]. To be effective, such prevention requires on one hand that pregnant women book ANC during the first trimester of pregnancy, and that ANC providers systematically screen women to identify those at a high risk of developing HDP, on the other hand. Our study found a high prevalence of late ANC booking (83.3%). This finding is consistent with a previous study from DRC in which the magnitude of late ANC booking was 82.4% [43], suggesting that late ANC booking is a public health issue in DRC. Surprisingly, women booking ANC beyond 20 weeks of pregnancy were more likely to be screened than those booking before 20 weeks. One possible explanation is that preeclampsia, the most prevalent HDP, arises after the 20th week of pregnancy [17]. Hence, ANC providers would be inadvertently more interested in screening women with a gestational age greater than 20 weeks, rather than those with a less advanced pregnancy.

Our findings alert health authorities to the low level of screening for HDP in Kinshasa. With such low provision of screening, it is not surprising that HDP remain a public health problem in Kinshasa [29, 44]. The study highlights the need for improving the provision of screening for HDP in order to contribute to reducing HDP-related morbidity and mortality.

To improve the screening for HDP in Kinshasa, interventions targeting both community and health system are needed. At the community level, pregnant women should be advised to book ANC in an earlier stage of pregnancy, in order to benefit from early screening for HDP [6]. At the health system level, there is a need to update national ANC guidelines and to train health providers accordingly. There is also a need to enhance the availability of urine tests for protein, and without any additional payment from women [45].

The main strength of this study is that data were collected during an actual ANC consultation, avoiding bias due to self-reporting or recall bias. To our knowledge, this is the first study in Kinshasa focusing on the screening for HDP during antenatal care.

Nonetheless, the study has some limitations, as the provision of antenatal service was assessed by direct observation of an antenatal consultation, health providers might have made an extra effort to give their best quality service at the time when the research team visited the clinic. We attempted to mitigate this Hawthorne effect by having data collectors stay of several days, which may have helped to reduce ANC provider awareness of the presence of the data collector.

## Conclusion

Our results show that screening for HDP during the first antenatal visit in Kinshasa is not universal. The gap in the quality of screening was in the identification of maternal risk factors, and a lack of urine testing for proteinuria. The factors associated with the screening included maternal as well as clinics’ characteristics. Our study highlights the need to improve the availability and provision of urine testing for proteinuria as well as risk factors assessment during the first ANC visit. More effort should also be made at the women level to increase early ANC booking.

## Data Availability

The datasets used during the current study are available from the corresponding author on reasonable request.

## Abbreviations

ACGO: American College of Obstetricians and Gynecologists
ANC: Antenatal care
AOR: Adjusted odds ratio
CI: confidence interval
DHS: Demographic and Health Survey
DRC: Democratic Republic of Congo
EmONC: emergency obstetric and neonatal care
GEE: generalized estimating equations
HDP: Hypertensive disorders in pregnancy
IQR: interquartile range
LMIC: Low- and Middle-Income Countries
NHIS: National Health Information System
NICE: National Institute for Health and Clinical Excellence
OR: Crude odds ratio
PHCs: Primary health centres
SARA: Service Availability and Readiness Assessment study
SD: Standard deviation
VIF: Variance inflation factor
